# Rare Oral Crohn’s Disease: A Case Report

**DOI:** 10.7759/cureus.39186

**Published:** 2023-05-18

**Authors:** Filipa Veiga, Paula Maria Leite, José Ferrão, Marcelo M Prates, Luís S Fonseca

**Affiliations:** 1 Oral Surgery, Centro Hospitalar Universitário de Lisboa Central, Lisbon, PRT

**Keywords:** orofacial disease, infliximab, oral pathology, chronic granulomatous disease, crohn’s disease

## Abstract

Crohn's disease is an inflammatory granulomatous and chronic disease characterized by inflammation of the gastrointestinal mucosa with extra-intestinal manifestations. Oral lesions seem to occur as specific lesions like lip swelling, cobblestone or tag lesions, or nonspecific lesions like ulcers.

The present case report describes an orofacial Crohn’s disease case, a rare presentation of Crohn’s disease, managed with infliximab.

Oral Crohn's disease refers to the spread of manifestations of Crohn’s disease and could precede other signs. Physicians have to be aware of oral mucosal changes. The treatment options are based on the use of corticosteroids, immune-modulators and biologics. The best plan and therapy to control oral Crohn's disease requires early and precise diagnosis.

## Introduction

Crohn's disease (CD) is an inflammatory and chronic disease characterized by inflammation of the orogastrointestinal mucosa. CD can affect any organ of the gastrointestinal system, from the mouth to the anus [1-4]. Its pathophysiology is still unknown, but it is believed to be multifactorial, including factors such as genetic, environmental, bacterial and autoimmune [2,4].

CD has increased its incidence and prevalence worldwide [5]. The onset of the initial presentation varies between the ages of 15 and 30 years old, but it can occur at any age [4,6].

The disease goes through periods of exacerbation and remission [7,8], and the clinical manifestations can be divided into intestinal and extraintestinal. In most cases, CD is a chronic and progressive disease [5]. The most common affected areas are the terminal ileum and colon. The oral cavity accounts for less than 5% of cases [8,9].

Extra-intestinal manifestations have a prevalence rate ranging from 16.7% to 40%, including classic manifestations such as arthropathy, dermatological, ocular and hepatobiliary diseases [4,6-8]. The prevalence of oral manifestations in CD ranges from 0.5% to 50% [1-4,6,7,10] and are more common in men and children [6,7].

In 60% of Crohn's disease patients, oral lesions may be the first sign. Although oral mucosal lesions and symptoms may be more severe during the period of disease flare, the correlation is not universal [2,7]. Specific oral lesions are characterized by the presence of noncaseating granuloma at the histopathology analysis. Specific lesions are less common than nonspecific lesions. The most frequent are indurated tag-like lesions, cobblestoning and mucogingivitis [7,11]. Non-specific lesions are usually secondary to a chronic inflammatory state, malnutrition and medication side effects. Aphthous stomatitis and pyostomatitis vegetans are the main nonspecific lesions [3,4,6,7,11].

The identification of their pathogenic mechanism and possibly those with precancerous potential is of tremendous importance in patient care [7,11].

Oral Crohn's disease (OCD) or Crohn's disease isolated only in the upper gastrointestinal tract is a relatively uncommon finding [1].

Management principles are similar to those with typical Crohn's disease, starting with topical therapy and progressing to systemic therapy such as glucocorticoids, 5-aminosalicylic acid, immunomodulators and biologics, like anti-tumor necrosis factor (TNF) antibodies [2,3,6,7,9,12]. The use of anti-TNF-α (infliximab in particular) has been suggested to have a role in the management of OCD and orofacial granulomatosis (OFG) in a number of case reports and case series [9,12,13].

In addition to pharmacotherapy, an appropriate diet, smoking cessation and prevention of infectious diseases are as well recommended [8]. Depression and anxiety, are factors that must also be treated, since they reduce the quality of life of these patients and make it difficult for therapeutic adherence [5].

This report describes a rare case of OCD managed with infliximab.

## Case presentation

**Figure 1:**
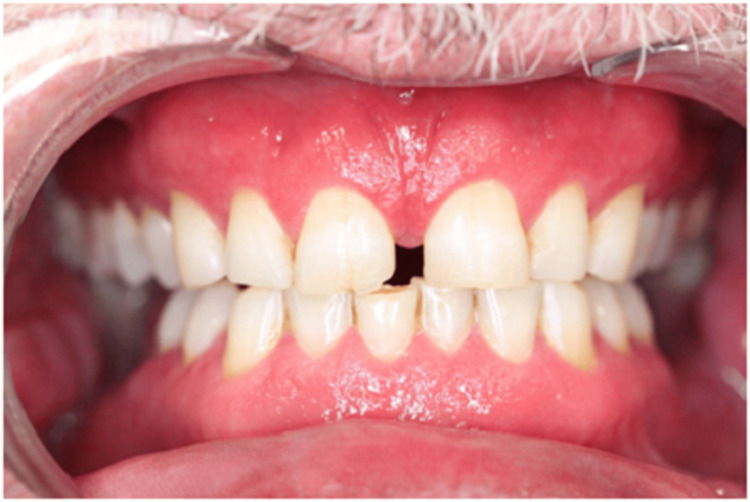
Muco-gingivitis.

**Figure 2:**
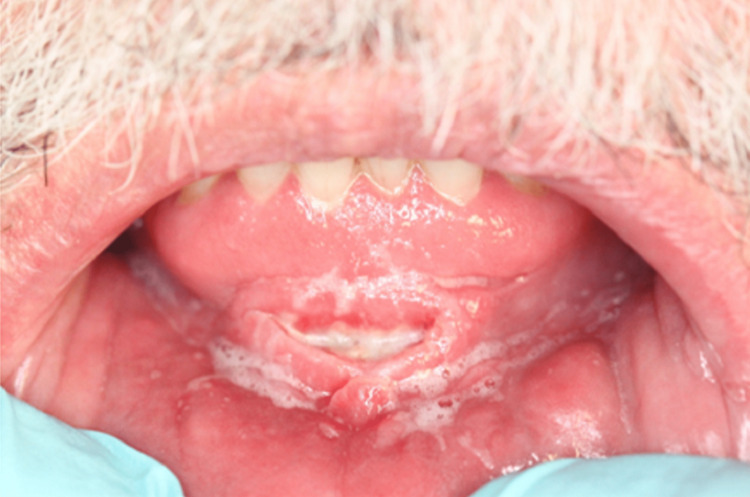
Hyperplastic lesion of the lower lip, buccogingival sulcus.

**Figure 3:**
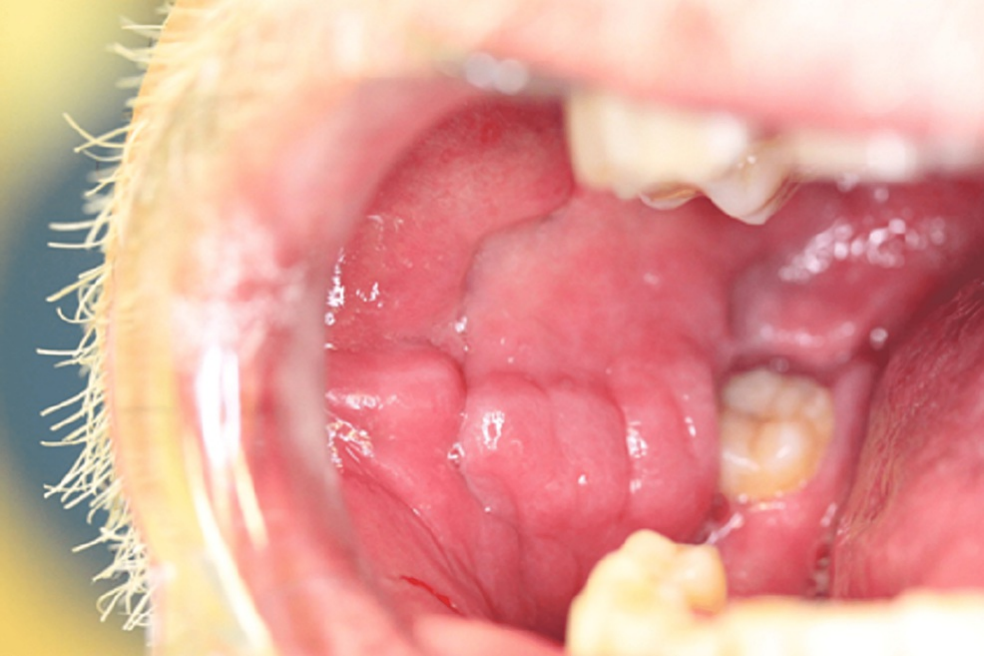
Cobblestones-like lesions of the right buccal mucosa.

A 61-year-old male patient, with a history of type two diabetes mellitus, systemic hypertension, hypercholesterolemia and transient ischaemic attack, was referred to the Stomatology Unit of Centro Hospitalar Universitário de Lisboa Central due to painful lesions on the oral mucosa. Preliminary examination revealed bilateral buccal mucosa and gingival erythema, ulceration on the lower labial frenulum and angular cheilitis. The lesions had been resolved with betamethasone 0.5 mg/mL mouth-rinsing, four times a day, during one week. One month after a slow withdrawal of the medication, the muco-gingivitis relapsed (Figure 1), a hyperplastic lesion of the lower lip mucosa had progressively appeared (Figure 2), such as some nodular cobblestone-like lesions of the buccal mucosa (Figure 3) and a swelling of the lower lip. 

**Figure 4:**
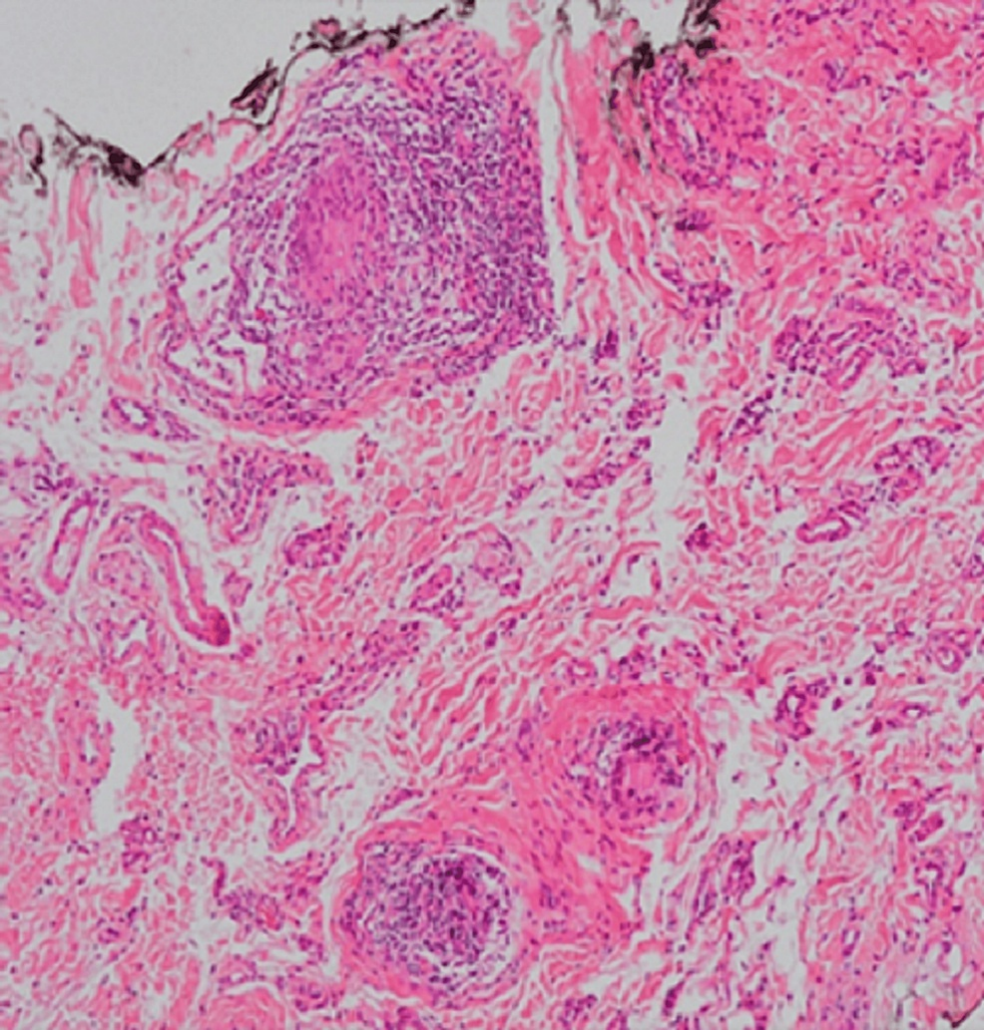
Lymphohistiocytic nodules surrounded by multinucleated giant cells, without well-formed epithelioid cell granulomas nor caseous necrosis in the subepithelial connective tissue. (H&E staining, 100X)

Samples of the suspicious lesions were collected by incisional biopsies. The histopathological evaluation, under periodic acid-Schiff (PAS), Grocott's methenamine silver staining and Ziehl-Neelsen (BAAR) protocols, was negative for microorganisms presence, neither foreign bodies under polarized light. The hematoxylin-eosin (HE) staining revealed vascular, hyperplastic fibrosis of the corion, lymphoplasmacytic and granulomatous inflammatory infiltration, with multinucleated giant cells. Neither well-formed epithelioid cell granulomas nor caseous necrosis were found (Figure 4).

Such histological findings reinforce the suspicion of a granulomatous disease, specifically CD.

The patient underwent a detailed blood panel and was referred to gastroenterology for further assessment. The laboratory results for C-reactive protein (CRP) and fecal calprotectin were 11.5mg/L (reference interval: <5mg/L) and 52ug/g (reference interval: <50ug/g), respectively. He tested positive for anti-Saccharomyces cerevisiae IgA antibodies (ASCA). The Interferon-Gamma Release Assay (IGRA) was negative, and other laboratory findings, including infectious serology were unremarkable. The urinalysis and the thorax radiography were normal.

**Figure 5:**
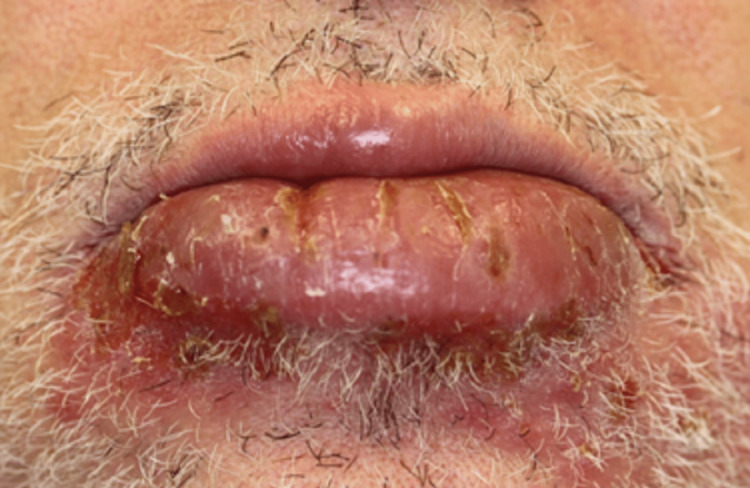
Swelling of lower lip with vertical fissures.

**Figure 6:**
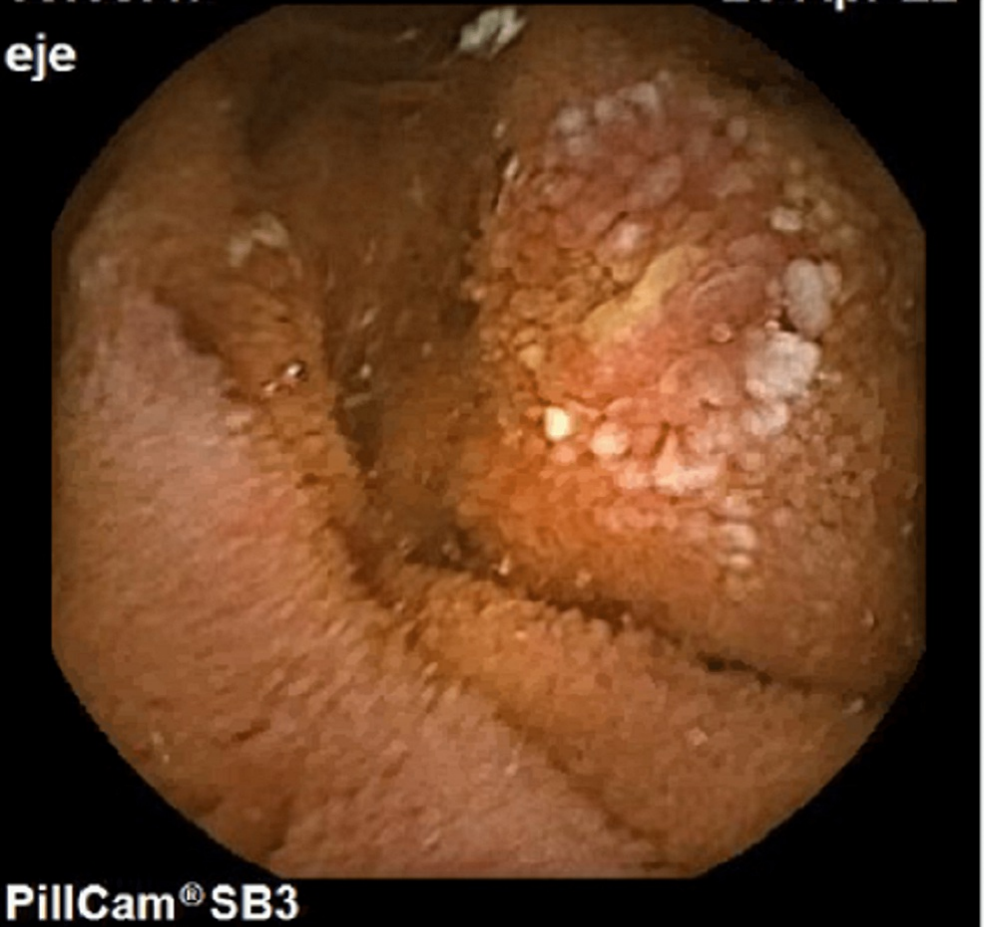
Capsule endoscopy: duodenal erosion.

The patient underwent a gastroenterologist evaluation and a colonoscopy was scheduled. Eight weeks later, the patient presented with a diffuse swelling of the lower lip with painful vertical fissures (Figure 5) and painful hyperplastic lesions of the right lower mucogengival sulcus and upper labial frenulum. He had no additional intestinal symptoms nor weight loss. Oral deflazacort, 30mg per day, and betamethasone 0.5mg/mL (mouth-rising, qid), were prescribed. The colonoscopy with ileoscopy was innocent, and the capsule endoscopy revealed a 3mm duodenal erosion with pearly base and hypertrophic edges (Figure 6).

Despite the absence of intestinal Crohn’s disease clinical and endoscopic diagnostic criteria, the OCD diagnosis was proposed, and a protocol with intravenous infliximab, 400mg, every eight weeks and methotrexate 10mg weekly, with folic acid supplementation, 5mg weekly, was started.

The biologic therapy was taken during 17 months without major complications and the exacerbations were reduced. The patient was advised to use betamethasone mouth-risings per need on flare-ups.

## Discussion

The oral mucosa is affected by a wide variety of systemic conditions due to the dynamic oral environment and the rapid cell turnover rate [2,3]. When faced with granulomatous lesions of the oral cavity, we should always consider many orofacial granulomatosis (OFG) [2,3,7,9,12]. In this patient, we excluded gastrointestinal symptoms and signs, foreign-body reactions, sarcoidosis, Wegener's granulomatosis and infectious granulomatous diseases such as tuberculosis, syphilis and fungal infections. The diagnosis of OCD was essentially clinical and reinforced by histopathologic study. The finding of non-caseating granulomas into oral biopsy, along with loose macrophage clusters, helped to define the diagnosis of OCD. Also, raised inflammatory markers and the positivity of the serological marker ASCA IgA are significantly associated with the diagnosis of CD [13].

In symptomatic cases, first-line treatment can often be non-specific with analgesia and topical or systemic corticosteroids [2,3,6,7,9,12]. In our patient, clinical remission was only achieved with infliximab, a biologic therapy.

In addition, during these 17 months, our patient did not present gastrointestinal manifestations. However, long-term follow-up of these patients would be wise. A proportion of patients who initially present features of OFG go on to develop gastrointestinal CD, indicating the need for close gastrointestinal clinical monitoring of such “OCD only” patients [9]. 

## Conclusions

OCD shows similar histopathological features to those observed in the intestinal mucosa and, despite the rare incidence of OCD, due to its clinical presentation, it should be included in the differential diagnosis of the granulomatous diseases of the oral mucosa. The therapy for OCD is not curative and relies on a variety of approaches to suppress inflammation and the mucosal immune response. The treatment with immunomodulators or anti-TNF-α, such as infliximab, might alter the course of the disease. The CD and the complications secondary to malnutrition or medication, due to their toxicity or immunosuppressive effect, can cause nonspecific and even underdiagnosed lesions, which require a multidisciplinary approach and therapeutic guidance.

In this case report, despite the patient only having oral manifestations, it is strongly recommended to have a frequent screening of intestinal lesions with a multidisciplinary approach.
